# The Relationship Between Hyperalbuminemia and Unscheduled Medical Visits: A Retrospective Cohort Study

**DOI:** 10.7759/cureus.65585

**Published:** 2024-07-28

**Authors:** Ryuichi Ohta, Kei Tsumura, Chiaki Sano

**Affiliations:** 1 Community Care, Unnan City Hospital, Unnan, JPN; 2 Family Medicine, Fuchu Hospital, Osaka, JPN; 3 Community Medicine Management, Shimane University Faculty of Medicine, Izumo, JPN

**Keywords:** rural, family medicine, general medicine, health care utilization, serum albumin, outpatient care, unscheduled medical visits, sympathetic nervous system, hyperalbuminemia

## Abstract

Introduction

Hyperalbuminemia, defined as elevated serum albumin levels, may influence healthcare utilization, particularly unscheduled medical visits. The sympathetic nervous system (SNS) regulates serum albumin, which is crucial for maintaining oncotic pressure and substance transport. SNS instability, linked to chronic diseases, can impact albumin levels. This study investigates the association between hyperalbuminemia and unscheduled medical visits in community hospital outpatient departments, aiming to establish its potential as a predictor of healthcare utilization.

Methods

This retrospective cohort study utilized electronic medical records from Unnan City Hospital, Japan, from September 2021 to August 2023. Participants were over 15 years old and had albumin data available, excluding those with acute albumin conditions. The case group consisted of 321 hyperalbuminemia patients (serum albumin ≥ 5 g/dL), matched monthly with 16 controls. Data on demographics, chronic diseases, and unscheduled medical visits were collected. Multivariate logistic regression analyzed the association between hyperalbuminemia and unscheduled medical visits.

Results

Among 716 participants, the hyperalbuminemia group (mean age 59.13 years) was younger than the non-hyperalbuminemia group (mean age 74.36 years). Hyperalbuminemia patients had a higher BMI, pulse rate, and prevalence of diabetes, dyslipidemia, and brain stroke. Significant associations were found between hyperalbuminemia and unscheduled medical visits (OR 2.35, 95% CI 1.56-3.53, p < 0.001), age, BMI, pulse rate, and brain stroke.

Conclusion

Hyperalbuminemia is significantly associated with increased unscheduled medical visits in rural outpatient settings. Routine serum albumin assessments can aid in risk stratification and personalized care, potentially reducing acute healthcare needs. Future research should explore underlying mechanisms and broader populations to enhance clinical applications.

## Introduction

In recent years, medical research has increasingly focused on understanding the complex interplay between physiological processes and healthcare utilization [1]. One area of interest is hyperalbuminemia, a condition characterized by elevated serum albumin levels, and its potential relationship with the frequency of unscheduled medical visits in outpatient departments of community hospitals [2,3]. Serum albumin, a predominant plasma protein in human blood, is crucial in maintaining oncotic pressure and transporting various substances throughout the body [4].

Numerous factors influence the production of serum albumin, including the state of the sympathetic nervous system. The sympathetic nervous system, an autonomic nervous system component, is instrumental in the body's fight-or-flight response and regulates various bodily functions [5,6]. Instability in this system, leading to the activation of sympathetic nerves, has been linked to the onset of various chronic diseases, such as hypertension, dyslipidemia, diabetes, insomnia, and brain stroke [7,8]. These associations highlight the importance of understanding the sympathetic nervous system's role in disease pathology.

Despite the acknowledged importance of the sympathetic nervous system in regulating various physiological processes, there is a significant gap in the current understanding of its role in hyperalbuminemia [9]. Hyperalbuminemia has been relatively under-investigated, particularly concerning its potential correlation with the use of unscheduled medical visits [10]. This lack of research presents a critical gap in medical knowledge, limiting our ability to fully understand and effectively address the health implications associated with abnormal serum albumin levels.

This study seeks to address this gap by exploring the relationship between hyperalbuminemia and the high usage of unscheduled medical visits in outpatient departments of community hospitals. Specifically, it aims to uncover how hyperalbuminemia is associated with increased healthcare utilization, providing insights into its potential as a marker for predicting the need for unscheduled medical care.

## Materials and methods

This retrospective cohort study aimed to identify the relationship between hyperalbuminemia and unscheduled medical visits affected by the instability of sympathetic nerve systems in rural hospitals. Using data from electronic medical records, multivariate logistic regression was performed with unscheduled medical visits as the dependent variable and hyperalbuminemia as the independent variable.

Setting

In 2022, Unnan City had a total population of 35,738, comprising 17,231 males and 18,507 females. Residents aged 65 years and older made up 40.27% of the population. The area was served by a single public hospital with 281 beds, categorized into 155 acute care, 48 general, 30 rehabilitation, and 48 chronic care beds. The Department of Family Medicine at this hospital coordinates the care of internal medicine patients in collaboration with various healthcare professionals [11].

Participants

Participants for the study were selected from patients who visited Unnan City Hospital in Unnan, Japan. The inclusion criteria were patients over 15 years old with available albumin data because they were cared for not by pediatrics but by internal medicine in Japan and considered to be developed as adults. Exclusion criteria were the lack of albumin data and conditions like dehydration, infection, or other inflammatory diseases that could acutely affect albumin levels through the review of the medical chart [12,13]. Patient data was extracted from the hospital's electronic medical records from September 2021 to August 2023. Throughout the study, cases with hyperalbuminemia were identified, totaling 321 cases in the case group. Correspondingly, the control group consisted of 16 cases selected each month based on consecutive hospital visits.

Data collection

Dependent Variable

As a primary outcome, an unscheduled medical visit was used to assess patients’ instability of medical conditions. In this research, an unscheduled medical visit was defined as an unscheduled medical visit for the care of new symptoms during the study duration. The measurement of the unscheduled medical visit started with the first visit to the hospital for regular check-ups for chronic diseases. This study collected data on the first unscheduled medical visits as a group of unscheduled medical visits.

Independent Variable and Covariates

This study designated hyperalbuminemia as the dependent variable, defined as a serum albumin level of ≥ 5 g/dL, following the criteria from previous research [14]. Factors related to hyperalbuminemia were identified based on earlier studies and were considered covariates; these covariates' data were extracted from electronic medical records [12,13]. The covariates included age, sex, height, weight, body mass index, and chronic diseases such as hypertension, dyslipidemia, diabetes, heart failure, cancer, and brain stroke. Patients lacking data on these variables were excluded from the study.

Analysis

For continuous variables, data normality was assessed before conducting statistical tests. Parametric data were analyzed using the Student’s t-test, while nonparametric data were evaluated with the Mann-Whitney U test. Categorical data were analyzed using the chi-squared test. For the logistic regression model, serum albumin levels were dichotomized into two categories: ≥ 5 g/dL and below 5 g/dL. A multivariate logistic regression analysis investigated the association between hyperalbuminemia and its related factors. The models included all variables linked to unscheduled medical visits as the dependent variable, with hyperalbuminemia as the independent variable, considering only those significant in univariate regression models of hyperalbuminemia. Data analyses were conducted using Easy R, version 1.23 (R Foundation for Statistical Computing, Vienna, Austria) [15]. Statistical significance was set at p<0.05.

Ethical consideration

The hospital was assured that patient anonymity and confidentiality would not be compromised. Information related to this study was posted on the hospital’s website without disclosing any patient details. In addition, the contact information of hospital personnel was posted on a website to address questions regarding this study. The Unnan City Hospital Clinical Ethics Committee approved the study protocol (no. 20230034).

## Results

Demographics of the participants

This study analyzed data from 716 participants, categorizing them based on their serum albumin levels, to investigate the relationship between hyperalbuminemia and the frequency of unscheduled medical visits in outpatient settings. The participants were divided into two groups: those with hyperalbuminemia (serum albumin levels ≥ 5 g/dL) and those without hyperalbuminemia. Key demographic and clinical characteristics were assessed and compared between these groups.

The mean age of the participants was 67.53 years (SD 16.74), with those in the hyperalbuminemia group being significantly younger (mean age 59.13 years, SD 14.54) compared to the non-hyperalbuminemia group (mean age 74.36 years, SD 15.25) (p < 0.001). Males constituted a larger proportion of the hyperalbuminemia group (64.2%) than the non-hyperalbuminemia group (49.6%) (p < 0.001). The mean body mass index (BMI) was higher in the hyperalbuminemia group (23.62, SD 4.81) compared to the non-hyperalbuminemia group (21.95, SD 4.36) (p < 0.001). Mean serum albumin levels were significantly elevated in the hyperalbuminemia group (5.13 g/dL, SD 0.17) compared to the non-hyperalbuminemia group (3.98 g/dL, SD 0.54) (p < 0.001). Regarding blood pressure, no significant difference was found in systolic blood pressure between the groups (p = 0.793). Still, diastolic blood pressure was higher in the hyperalbuminemia group (80.58 mmHg, SD 14.12) compared to the non-hyperalbuminemia group (70.14 mmHg, SD 13.22) (p < 0.001). Pulse rates were also higher in the hyperalbuminemia group (79.36 bpm, SD 17.02) compared to the non-hyperalbuminemia group (70.55 bpm, SD 13.83) (p < 0.001).

A significant finding was the higher frequency of unscheduled medical visits in the hyperalbuminemia group (47.7%) compared to the non-hyperalbuminemia group (30.4%) (p < 0.001). This suggests a notable association between elevated serum albumin levels and increased healthcare utilization for unscheduled medical needs.

The prevalence of certain chronic conditions differed between the two groups. Diabetes was significantly more common in the hyperalbuminemia group (35.2%) compared to the non-hyperalbuminemia group (1.8%) (p < 0.001). Similarly, dyslipidemia was more prevalent in the hyperalbuminemia group (26.8%) compared to the non-hyperalbuminemia group (13.2%) (p < 0.001). The occurrence of brain stroke was also higher in the hyperalbuminemia group (7.5%) than in the non-hyperalbuminemia group (1.8%) (p < 0.001) (Table 1).

**Table 1:** Demographic data of the participants with the categorization of the level of serum albumin BMI: body mass index; SD: standard deviation

Factor	Total	Hyperalbuminemia	No hyperalbuminemia	p-value
n	716	321	395	
Age, year old, mean (SD)	67.53 (16.74)	59.13 (14.54)	74.36 (15.25)	<0.001
Male sex, n (%)	402 (56.1)	206 (64.2)	196 (49.6)	<0.001
BMI, mean (SD)	22.69 (4.64)	23.62 (4.81)	21.95 (4.36)	<0.001
Albumin, mean (SD)	4.50 (0.71)	5.13 (0.17)	3.98 (0.54)	<0.001
Systolic blood pressure, mean (SD)	136.53 (53.48)	137.11 (20.98)	136.06 (69.52)	0.793
Diastolic blood pressure, mean (SD)	74.82 (14.58)	80.58 (14.12)	70.14 (13.22)	<0.001
Pulse, mean (SD)	74.50 (15.95)	79.36 (17.02)	70.55 (13.83)	<0.001
Unscheduled medical visit, n (%)	273 (38.1)	153 (47.7)	120 (30.4)	<0.001
Hypertension, n (%)	250 (34.9)	117 (36.4)	133 (33.7)	0.478
Diabetes, n (%)	120 (16.8)	113 (35.2)	7 (1.8)	<0.001
Dyslipidemia, n (%)	138 (19.3)	86 (26.8)	52 (13.2)	<0.001
Heart failure, n (%)	102 (14.2)	47 (14.6)	55 (13.9)	0.83
Brain stroke, n (%)	31 (4.3)	24 (7.5)	7 (1.8)	<0.001
Cancer, n (%)	106 (14.8)	53 (16.5)	53 (13.4)	0.29

Multivariate logistic regression analysis

A multivariate logistic regression analysis was performed to identify factors independently associated with unscheduled medical visits. The analysis revealed that hyperalbuminemia was a significant predictor of unscheduled medical visits (odds ratio [OR] 2.35, 95% confidence interval [CI] 1.56-3.53, p = 0.000044). Other significant predictors included age (OR 1.02, 95% CI 1.01-1.03, p = 0.0012), BMI (OR 0.93, 95% CI 0.89-0.97, p = 0.00057), pulse rate (OR 1.03, 95% CI 1.02-1.04, p < 0.0001), and the presence of a brain stroke (OR 3.14, 95% CI 1.36-7.28, p = 0.0075) (Table 2).

**Table 2:** The multivariate logistic regression model for unscheduled medical visits with hyperalbuminemia and other covariates The definition of hyperalbuminemia was serum albumin level ≧ 5 g/dL; BMI: body mass index

Factor	Odds ratio	95%CI	p-value
Hyperalbuminemia	2.35	1.56-3.53	<0.001
Age	1.02	1.01-1.03	0.0012
Male sex	1.09	0.78-1.55	0.61
Body mass index	0.93	0.89-0.97	<0.001
Systolic blood pressure	1	1.00-1.00	0.58
Diastolic blood pressure	1.01	1.00-1.02	0.17
Pulse rate	1.03	1.02-1.04	<0.001
Dyslipidemia	0.7	0.45-1.07	0.1
Brain stroke	3.14	1.36-7.28	0.0075
Cancer	1.23	0.79-1.94	0.36
Heart failure	1.31	0.82-2.11	0.26

## Discussion

Our study contributes to understanding the complex interplay between hyperalbuminemia and healthcare utilization patterns in a rural outpatient setting. Serum albumin, primarily produced by the liver, is crucial in maintaining oncotic pressure and transporting various substances, including hormones and drugs, throughout the body. The elevation of serum albumin levels, defined in our study as ≥ 5 g/dL, typically reflects good nutritional status and overall health [16]. However, in the context of chronic diseases such as diabetes and dyslipidemia, which were more prevalent among our hyperalbuminemia cohort, the interpretation of elevated serum albumin levels becomes more nuanced [17]. Through this study, higher serum albumin may show better health conditions, but high serum albumin should be recognized based on nutrition and patients' physical and psychological stress.

The sympathetic nervous system (SNS), implicated in regulating albumin synthesis through its influence on liver function, may offer mechanistic insights into our findings [18,19]. Activation of the SNS, often seen in chronic stress conditions and various chronic diseases, could stimulate liver function and increase serum albumin production [20]. This hypothesis aligns with studies linking sympathetic activation to adverse health outcomes such as hypertension, dyslipidemia, and diabetes, all of which were more prevalent in our hyperalbuminemia group [21]. Persistent activation of SNS can gradually increase serum albumin, as even patients with poor nutritional conditions can show apparent normal albumin levels [21]. Thus, while hyperalbuminemia may indicate physiological resilience in some contexts, its association with increased healthcare utilization suggests underlying health complexities and poor nutritional conditions that warrant closer clinical attention.

Identifying hyperalbuminemia as a predictor of unscheduled medical visits carries significant implications for clinical practice. Healthcare providers in outpatient settings, particularly in rural or resource-limited environments, often face challenges in managing patient flow and optimizing healthcare resource allocation [22,23]. By recognizing hyperalbuminemia as a potential marker for increased healthcare needs, clinicians can proactively monitor and manage patients with elevated serum albumin levels, especially those with concurrent chronic conditions. Patients with hyperalbuminemia can have a high risk of unscheduled medical visits, so physicians can take care of their subtle changes in symptoms and discuss self-management and help-seeking behaviors for their symptoms in depth, which can prevent the frequency of their unscheduled medical visits [24].

Moreover, integrating serum albumin assessment into routine clinical practice could enhance risk stratification and guide personalized care plans. For instance, patients identified with hyperalbuminemia might benefit from more frequent follow-ups, preventive health screenings, and targeted interventions aimed at managing underlying chronic diseases [25]. Although there was no significant relationship between unscheduled medical usage and chronic diseases except for brain stroke, chronic disease can trigger brain stroke and cardiovascular diseases [25]. This proactive approach improves patient outcomes and optimizes healthcare resource utilization by mitigating the risk of acute exacerbations and hospitalizations, which can improve their health conditions and quality of life.

Our findings corroborate and extend existing literature on the prognostic significance of serum albumin levels across various disease states. Previous studies have highlighted the predictive value of hypoalbuminemia for adverse outcomes such as mortality and morbidity in conditions like chronic kidney disease and heart failure [26-28]. Conversely, less attention has been paid to hyperalbuminemia and its implications for healthcare utilization, particularly in outpatient settings. This study shows that the abnormality of serum albumin should be carefully considered when predicting patients in general medicine because general physicians can approach multiple patients with vague symptoms. Their serum albumin can alarm general physicians regarding the intensity of medical investigation.

Our study fills a critical gap in the literature by demonstrating a clear association between elevated serum albumin levels and increased unscheduled medical visits. It underscores the need for further research to elucidate the underlying mechanisms driving this association and explore potential interventions to mitigate healthcare utilization among high-risk populations identified through serum albumin assessment. In addition, hyperalbuminemia may affect various health conditions, causing healthcare complications from help-seeking behaviors, except for unscheduled medical visits because of hypersympathetic nerve stimulation [29,30]. The following studies can investigate the relationship between hyperalbuminemia conditions and other help-seeking behaviors in patients.

Despite its contributions, our study has several limitations that merit consideration. The retrospective cohort design limits our ability to establish causality between hyperalbuminemia and unscheduled medical visits. Future prospective studies are warranted to validate our findings and elucidate the temporal relationship between serum albumin levels and healthcare utilization patterns. Additionally, our study was conducted in a single rural hospital setting with specific demographic characteristics, potentially limiting the generalizability of our findings to other healthcare settings and patient populations. Future research should include diverse patient cohorts from urban and suburban settings to enhance the external validity of our results. Furthermore, the exclusion criteria related to acute inflammatory conditions and other confounding factors may have influenced the composition of our study population and the observed prevalence of hyperalbuminemia. Future studies should consider adjusting for additional covariates and exploring subgroup analyses to better understand the differential impact of hyperalbuminemia across different patient profiles.

## Conclusions

Our study highlights hyperalbuminemia as a clinically relevant biomarker for predicting increased healthcare utilization in rural outpatient settings. By incorporating serum albumin assessment into routine clinical practice, healthcare providers can enhance risk stratification, optimize resource allocation, and improve patient outcomes. Future research should focus on elucidating the mechanistic underpinnings of hyperalbuminemia’s association with healthcare utilization and exploring targeted interventions to mitigate its impact on patient care. These efforts will pave the way for personalized medicine approaches to optimize healthcare delivery and improve patient outcomes across diverse healthcare settings.
